# Thrombotic and haemorrhagic complications in critically ill patients with COVID-19: a multicentre observational study

**DOI:** 10.1186/s13054-020-03260-3

**Published:** 2020-09-18

**Authors:** Akshay Shah, Killian Donovan, Anna McHugh, Manish Pandey, Louise Aaron, Charlotte A. Bradbury, Simon J. Stanworth, Raza Alikhan, Stephen Von Kier, Keith Maher, Nicola Curry, Susan Shapiro, Matthew J. Rowland, Matt Thomas, Richard Mason, Matthew Holland, Tom Holmes, Michael Ware, Stefan Gurney, Stuart R. McKechnie

**Affiliations:** 1 Radcliffe Department of Medicine, Level 4 Academic Block, University of Oxford, John Radcliffe Hospital, Headley Way, Oxford, OX3 9DU UK; 2 Adult Intensive Care Unit, John Radcliffe Hospital, Oxford University Hospitals NHS Foundation Trust, Oxford, UK; 3 Intensive Care Unit, North Bristol NHS Trust, Bristol, UK; 4 Adult Intensive Care Unit, University Hospital of Wales, Cardiff, Wales, UK; 5 Faculty of Health Sciences, University of Bristol, Bristol, UK; 6 Haematology Theme, NIHR Oxford Biomedical Research Centre, Oxford, UK; 7 Haemostasis and Thrombosis, Department of Haematology, University Hospital of Wales, Cardiff, UK; 8 Blood Management and Conservation Service, Oxford University Hospitals NHS Foundation Trust, Oxford, UK; 9 Oxford Haemophilia & Thrombosis Centre, Department of Haematology, Churchill Hospital, Oxford University Hospitals NHS Foundation, Oxford, UK; 10 Kadoorie Centre for Critical Care Research, Nuffield Department of Clinical Neurosciences, University of Oxford, Oxford, UK; 11 Intensive Care Unit, Bristol Royal Infirmary, University Hospitals Bristol NHS Trust, Bristol, UK

**Keywords:** COVID-19, Thrombosis, Haemorrhage, Heparin

## Abstract

**Background:**

Optimal prophylactic and therapeutic management of thromboembolic disease in patients with COVID-19 remains a major challenge for clinicians. The aim of this study was to define the incidence of thrombotic and haemorrhagic complications in critically ill patients with COVID-19. In addition, we sought to characterise coagulation profiles using thromboelastography and explore possible biological differences between patients with and without thrombotic complications.

**Methods:**

We conducted a multicentre retrospective observational study evaluating all the COVID-19 patients received in four intensive care units (ICUs) of four tertiary hospitals in the UK between March 15, 2020, and May 05, 2020. Clinical characteristics, laboratory data, thromboelastography profiles and clinical outcome data were evaluated between patients with and without thrombotic complications.

**Results:**

A total of 187 patients were included. Their median (interquartile (IQR)) age was 57 (49–64) years and 124 (66.3%) patients were male. Eighty-one (43.3%) patients experienced one or more clinically relevant thrombotic complications, which were mainly pulmonary emboli (*n* = 42 (22.5%)). Arterial embolic complications were reported in 25 (13.3%) patients. ICU length of stay was longer in patients with thrombotic complications when compared with those without. Fifteen (8.0%) patients experienced haemorrhagic complications, of which nine (4.8%) were classified as major bleeding. Thromboelastography demonstrated a hypercoagulable profile in patients tested but lacked discriminatory value between those with and without thrombotic complications. Patients who experienced thrombotic complications had higher D-dimer, ferritin, troponin and white cell count levels at ICU admission compared with those that did not.

**Conclusion:**

Critically ill patients with COVID-19 experience high rates of venous and arterial thrombotic complications. The rates of bleeding may be higher than previously reported and re-iterate the need for randomised trials to better understand the risk-benefit ratio of different anticoagulation strategies.

**Graphical abstract:**

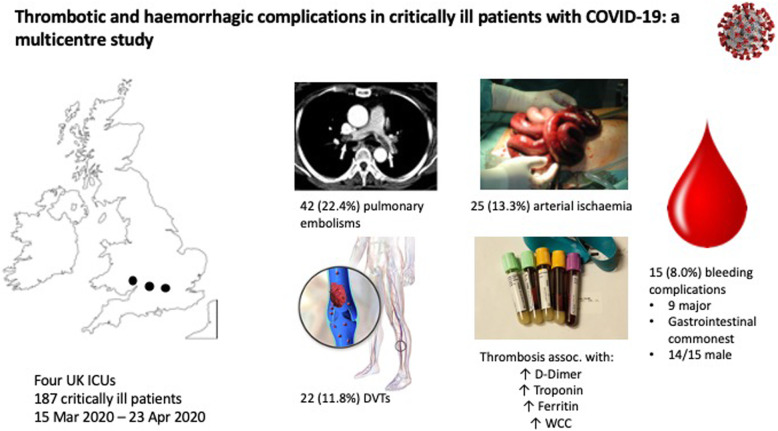

## Background

Optimal management of thrombotic complications that are recognised as a common feature of coronavirus disease 2019 (COVID-19) remains a major clinical challenge in intensive care units (ICUs) [1–3]. Proposed underlying mechanisms include an excessive immune response cytokine storm, endotheliopathy, intussusceptive angiogenesis and hypercoagulability [1, 4]. Neutrophilia and elevated inflammatory and coagulation markers, such as D-dimer and fibrinogen have been associated with increased mortality in COVID-19 [5, 6].

The reported incidence of thromboembolic complications in ICU patients with COVID-19 ranges from 21 to 69%, but these data are predominantly from single-centre studies [7–11]. Current research priorities include the optimal agent, dose and duration for prophylaxis and treatment of thrombosis, identification of clinical characteristics or biomarkers to aid risk stratification and the potential role of viscoelastic tests of coagulation [1, 12, 13]. In addition, bleeding rates have been sparsely reported, which is problematic, particularly as recent guidelines recommend applying higher dose low molecular weight heparin (LMWH) thromboprophylaxis [13, 14].

The objectives of our study were to (i) define the incidence of thrombotic and haemorrhagic complications in COVID-19 patients admitted to four ICUs in the United Kingdom (UK) and (ii) identify possible differences in ICU admission laboratory, coagulation and thromboelastography profiles between patients who develop thrombotic complications compared with those that do not.

## Methods

We report our findings in accordance with the Strengthening the Reporting of Observational Studies in Epidemiology (STROBE) statement [15] (Additional file 1).

### Study design and setting

We conducted a retrospective, observational study of all patients admitted to adult intensive care units (ICUs) in four tertiary UK hospitals (John Radcliffe Hospital, Oxford; Southmead Hospital and Bristol Royal Infirmary, Bristol; and University Hospital of Wales, Cardiff) between March 15, 2020, and May 05, 2020, with suspected or laboratory-confirmed SARS-CoV-2 infection. There were no exclusion criteria. Laboratory confirmation of SARS-CoV-2 infection was defined as a positive result of real-time reverse-transcriptase polymerase chain reaction (RT-PCR) assay of the upper respiratory tract (nasopharyngeal and oropharyngeal) and/or lower respiratory tract (sputum, endotracheal aspirate, or bronchoalveolar lavage) lavage. Suspected SARS-CoV-2 cases included patients with a clinical history and features of COVID-19 with radiological lesions compatible with severe COVID-19, despite a negative RT-PCR test. These patients were admitted to dedicated COVID ICUs in all centres and were not managed differently to those with laboratory-confirmed infection.

As part of standard care at all institutions, all ICU admissions received standard weight-based low molecular weight heparin (LWMH) thromboprophylaxis. Details of each site’s thromboprophylaxis protocol are available in Additional file 2. Therapeutic anticoagulation with LWMH was commenced for image-proven thrombosis or when there was a strong clinical suspicion and imaging studies were difficult to perform because of illness severity or infection control measures. Routine monitoring of anti-Xa levels in patients on therapeutic LWMH was not performed unless there was a specific clinical indication such as severe renal impairment, coagulopathy or active bleeding. It is worth noting the correlation between anti-Xa levels and thromboembolic and bleeding events is very weak [16]. Patients receiving therapeutic intravenous unfractionated heparin infusions were monitored using anti-Xa levels rather than activated partial thromboplastin times. The initial target range was 0.3 to 0.7 units/mL but was increased to 0.5 to 0.8 units/mL in the event of haemofiltration circuit thrombosis. Levels were checked 6 h after a rate change. Once daily monitoring was acceptable if the infusion rate was stable with two stable consecutive 6-h anti-Xa levels.

CT pulmonary angiograms (CTPAs) and lower limb Doppler ultrasound scans were performed at the discretion of the treating clinician. Common indications for CTPA included worsening oxygenation, despite maximal support therapies such as proning and inhaled nitric oxide, haemodynamic instability with evidence of right ventricular impairment or extremely high D-Dimer values. No centre routinely screened for deep vein thrombosis.

Research ethics committee approval was not required for this study as per the UK Health Research Authority Decision tool (http://www.hra-decisiontools.org.uk/research/). The study was deemed to be service evaluation at each site and the need for consent was waived. No direct patient identifiable data were collected.

### Outcomes

Our primary outcome of interest was the occurrence of any thrombotic complication (pulmonary embolism, deep venous thrombosis, peripheral arterial ischaemia, myocardial infarction, stroke or extracorporeal circuit thrombosis). Secondary outcomes included haemorrhagic complications and the results of coagulation and thromboelastography tests. The number of patients who had died, had been discharged, and were still admitted in the ICU as of May 15, 2020, were recorded. ICU length of stay also was determined. The shortest follow-up period was 10 days. Baseline characteristics of our study cohort were compared with UK national data collected on ICU patients with COVID-19 by the Intensive Care National Audit and Research Centre [17].

### Data collection and definitions

Anonymised case data on clinical characteristics, patient demographics, comorbidities and results of imaging studies were extracted from routinely collected healthcare records. Standard laboratory and coagulation parameters measured at, or within 48 h of, ICU admission (whichever came first) were extracted for all patients. In addition, we also captured thromboelastography data at, or within 48 h of, ICU admission for twenty patients. Additional coagulation results were extracted in patients who experienced bleeding complications. Anticoagulation regime at the time of ICU admission was also recorded.

Bleeding severity and disseminated intravascular coagulation (DIC) scoring were assessed using recognised definitions. Major bleeding was defined as (i) fatal bleeding and/or; (ii) symptomatic bleeding in a critical area or organ, such as intracranial, intraspinal, intraocular, retroperitoneal, intraarticular or pericardial, or intramuscular with compartment syndrome and/or; (iii) bleeding causing a fall in haemoglobin level of 2 g/dL (1.24 mmol/L) or more or leading to transfusion of two or more units of whole blood or red blood cells [18]. Laboratory coagulation results at the closest time point to the onset of the index bleeding episode were extracted. Overt DIC was 5 points or more [19]. Thromboelastography was performed using the TEG6s system (Haemonetics Limited, UK).

### Statistical methods

No sample size calculation was performed for this study. Means for continuous variables were compared using independent group *t* tests when the data were normally distributed; otherwise, the Mann-Whitney test was used. Proportions for categorical variables were compared using the *χ*^2^ test. All statistical tests were two-tailed, and statistical significance was defined as *p* < 0.05. Analyses were performed using R version 3.2.4 (the R foundation for statistical computing).

## Results

### Demographics and clinical characteristics

We included 187 patients. The median (interquartile (IQR)) age was 57 (49–64) years and 124 (66.3%) patients were male. Of all included patients, 142 (77%) were either very fit or well according to the Clinical Frailty Scale [20]. Respiratory failure requiring invasive mechanical ventilation occurred in 167 (89.3%) patients. Other relevant patient characteristics, co-morbidities and ICU resource requirements are summarised in Table 1, alongside a comparison of UK national data.

**Table 1:** Clinical characteristics and outcomes of the study population (*n* = 187)

Characteristic	Study cohort	ICNARC comparator as of May 22, 2020 (***n*** = 9026)
**Age (years)**, median (IQR)	57 (49–64)	60 (51–67)
**Sex,** ***n*** **(%)**		
Male	124 (66.3)	6403 (71.0)
Female	63 (33.7)	2619 (29.0)
**Time period between symptom onset and hospital admission (days)**, median (IQR)	7 (5–9)	–
**APACHE II score**		
Mean (SD)	13.8 (6.3)	14.7 (5.3)
Median (IQR)	13 (10–13)	14 (11–18)
**PaO**_**2**_**/FiO**_**2**_ **ratio (mmHg),** median (IQR)	135 (103–182)	118.5 (84.7–165)
**PaO**_**2**_**/FiO**_**2**_ **ratio,** *n* (%)		
< 100 mmHg (< 13.3 kPa)	45 (24.0)	2982 (36.8)
100–200 mmHg (13.3–26.6 kPa)	105 (56.1)	3961 (48.9)
≥ 200 mmHg (≥ 26.7 kPa)	37 (19.8)	1161 (14.3)
**Body mass index (kg m**^**−2**^**)**	28 (25–32)	–
**Categories,** *n* (%)		
< 18.5	2 (1.1)	56 (0.7)
18.5 < 25	50 (26.7)	2118 (25.4)
25< 30	65 (34.7)	2932 (35.1)
30 < 40	53 (28.3)	2595 (31.1)
40+	14 (7.4)	643 (7.7)
**Ethnicity,** *n* (%)		
White	141 (76.6)	5468 (66.8)
Asian	19 (10.3)	1245 (15.2)
Black	16 (8.7)	797 (9.7)
Other	8 (4.3)	537 (6.6)
**Co-morbidities**, *n* (%)		
Hypertension	71 (43.1)	–
Diabetes	54 (30.2)	–
Ischaemic heart disease	16 (8.4)	–
Previous stroke	5 (3.6)	–
COPD or asthma	38 (24.2)	–
Previous PE/DVT	7 (5.5)	–
Malignancy	15 (6.4)	–
Chronic kidney disease	12 (8.2)	–
None	61 (32.6)	–
**Clinical Frailty Scale**, *n* (%)		
1–2	142 (77)	–
3–4	36 (19)	–
5+	7 (4)	–
**Advanced cardiovascular support**, *n* (%)	47 (25.1)	2119 (28.8)
**Advanced renal support**, *n* (%)	80 (42.8)	1848 (25.2)
**Respiratory support**, *n* (%)		
Non-invasive ventilation	42 (22.5)	
Invasive ventilation	167 (89.3)	5330 (72.5)
Prone position	101 (54.0)	–
ECMO	5 (2.7)	–
**No. of patients with thrombotic complications**, *n* (%)	**81 (43.3)**	**–**
Pulmonary embolism	42 (22.5)	–
Deep vein thrombosis	22 (11.8)	–
Arterial complications		–
Arterial ischaemia (peripheral or intestinal)	12 (6.4)	–
Cerebrovascular accident	8 (4.3)	–
Myocardial infarction	5 (2.7)	–
Extracorporeal circuit disruption	23 (12.3)	–
**ICU outcomes, as of May 15, 2020**, *n* (%)		
Died in ICU	59 (31.6)	3302 (44.3)
Still alive in ICU	33 (17.6)	–
Discharged from ICU	95 (50.8)	4145 (55.7)

*APACHE* Acute Physiology and Chronic Health Evaluation, *COPD* chronic obstructive pulmonary disease, *DVT* deep vein thrombosis, *ECMO* extracorporeal membrane oxygenation, *ICU* intensive care unit, *PE* pulmonary embolism

### Thrombotic and haemorrhagic complications

**Fig. 1:**
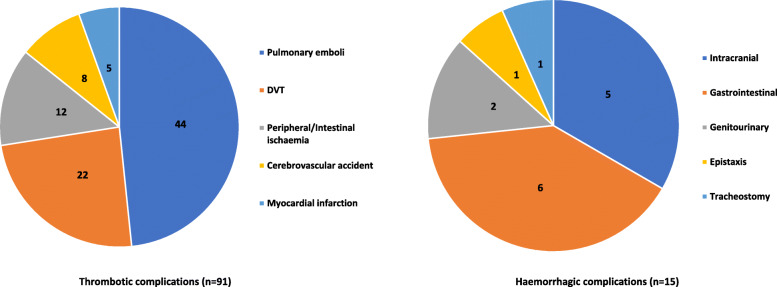
Description of thrombotic and haemorrhagic complications

Eighty-one (43.3%) patients experience one or more clinically relevant thrombotic complications (Table 1), which were predominantly pulmonary embolism or deep vein thrombosis (Fig. 1). Twenty-five (13.3%) patients experienced arterial complications, of which twelve involved peripheral or intestinal ischaemia (Fig. 1). One hundred CT pulmonary angiograms (CTPA) were performed of which 42 were positive for segmental or subsegmental pulmonary embolism. One hundred seventy-eight patients (95.1%) were already on either prophylactic or therapeutic anticoagulation (Table 2).

**Table 2:** Clinical characteristics, laboratory parameters and coagulation profiles among patients who experienced bleeding complications

Characteristic	***n*** = 15
**Age (years)**, median (IQR)	60 (51–71)
**Sex**, *n* (%)	
Male	14 (93.3)
Female	1 (6.7)
**Admission APACHE II score,** median (IQR)	14 (9–16)
**Bleeding complications**, *n* (%)	15 (8.0)
Gastrointestinal	6 (3.2)
Intracranial	5 (2.7)
Other (epistaxis (*n* = 1), Tracheostomy (*n* = 1), GU (*n* = 2))	4 (2.1)
**Severity of bleeding,** *n* (%)	
Major	9 (60)
Non-major	6 (40)
**Anticoagulation**, *n* (%)	
Prophylactic	8 (53.3)
Therapeutic	5 (33.3)
Data not available	2 (13.4)
**Time interval between ICU admission and bleeding episode (days),** median (IQR)	15 (6–25)
**No. requiring red blood cell transfusion,** *n* (%)	7 (46.7)
**SOFA score at time of bleed,** median (IQR)	9 (5–12)
**Organ support requirement (*n*** **= 12),** n	
0–1 advanced organ support requirement	6
> 1 advanced organ support requirement	6
**Laboratory data at time of bleeding episode (*n*** **= 12)**	
Platelet count (× 10^9^/L), median (IQR)	244 (163–325)
Prothrombin time (seconds)**,** median (IQR)	11.3 (10.6–11.8)
Activate partial thromboplastin time (seconds), median (IQR)	27.2 (23.6–34.2)
Fibrinogen (g/L), median (IQR)	4.8 (2.8–6.3)
**ICU length of stay (days),** median (IQR)	20 (10–35)

*APACHE* Acute Physiology and Chronic Health Evaluation, *GU* genitourinary, *ICU* intensive care unit, *SOFA* Sequential Organ Failure Assessment,

Fifteen patients (8.0%) experienced haemorrhagic complications, of which nine (4.8%) were classified as major bleeding (Table 2). Nearly all patients were male (*n* = 14) and gastrointestinal bleeding was the commonest site. Bleeding occurred at a median (IQR) of 15 (6–25) days following ICU admission. Seven patients required allogeneic red blood cell transfusions. At the time of bleeding episode, the median (IQR) sequential organ failure assessment (SOFA) was 9 (5–12) and five patients were established on therapeutic anticoagulation. The other four patients who experienced major bleeding were on standard thromboprophylaxis. No patients had overt DIC or a fibrinogen concentration of less than 1.5 g/L. Thrombotic and haemorrhagic complications for each participating centre are displayed in Additional file 3.

### Mortality

Overall ICU mortality was 31.6%. A higher proportion of patients with thrombotic complications died when compared with those without but this was not statistically significant (32 (39.5%) vs. 27 (25.5%), *p* = 0.059). Median (IQR) ICU length of stay was longer in patients who developed thrombotic complications 17 (11–27) days vs. 12 (7–13) days, *p* = 0.003).

### Laboratory and thromoboelastography markers

Patients who developed thrombotic complications had significantly higher D-Dimer (*p* < 0.001), troponin T (*p* = 0.008), troponin I (*p* < 0.001), white cell count (*p* = 0.024) and ferritin concentrations (*p* = 0.008) at ICU admission, when compared with those without thrombotic complications (Table 3). Thromboelastography data were available for twenty patients and suggested a hypercoagulable profile characterised by mean Alpha angle and maximal amplitude (MA) values at or above the upper limits of the normal reference range, extremely low LY30 values and higher fibrin contribution toward clot formation relative to platelets. However, we observed no differences in any of these parameters between both groups.

**Table 3:** Clinical characteristics, laboratory parameters and coagulation profiles among patients with and without thrombotic complications

Characteristic	All patients (***n*** = 187)	Thrombotic complication (***n*** = 81)	No thrombosis (***n*** = 106)	*p* value
**Age (years),** median (IQR)	57 (49–64)	59 (53–66)	56 (48–63)	0.046
**Sex,** *n* (%)				
Female	63 (32)	21 (26)	42 (40)	0.071
Male	124 (68)	60 (74)	64 (60)	–
**APACHE II score**, median (IQR)	13 (10–13)	14 (11–18)	13 (9–16)	0.594
**ISTH DIC score,** median (IQR)	3 (2–3)	3 (2–3)	2 (2–3)	
**Baseline treatments** (*n* = 185), *n* (%)				
Prophylactic LMWH or UFH, *n*	151	59 (31.3)	92 (49.9)	
Therapeutic LMWH or UFH, *n*	27	18 (9.9)	9 (5.0)	
Vitamin K antagonist, *n*	2	0 (0)	2 (1.1)	
Directly acting oral anticoagulant, *n*	2	0 (0)	2 (1.1)	
No anticoagulant, *n*	3	3 (1.7)	0	
**Laboratory parameters (approximate normal range)**				
Haemoglobin (g/L) (120–150)	121.1 (20.4)	121.2 (22.9)	121.0 (18.2)	0.948
Platelet count (×10^9^/L) (150–400)	241 (186–318)	238 (179–319)	243 (193–311)	0.749
White cell count (×10^9^/L) (4.0–11.0)	9.18 (6.81–12.43)	9.72 (7.82–12.64)	8.16 (6.02–11.55)	0.024
Lymphocyte count (×10^9^/L) (1.0–4.0)	0.80 (0.50–1.10)	0.80 (0.50–1.10)	0.80 (0.56–1.10)	0.321
Lactate dehydrogenase	629 (418–927)	700 (437–1032)	579 (415–820)	0.096
Peak troponin T (ng/mL) (*n* = 62)	27 (12–57)	44 (23–66)	18 (12–39)	0.008
Peak troponin I (ng/L) (*n* = 103)	14 (6–61)	26 (9–194)	9 (4–32)	< 0.001
CRP (mg/L) (0–5)	202 (128–294)	209 (143–300)	187 (121–267)	0.055
Ferritin (mcg/l) (10–200)	1126 (495–1880)	1305 (737–2301)	886 (370–1499)	0.008
**Coagulation parameters**				
Prothrombin time (seconds)	12.4 (11.0–14.9)	12.0 (11.0–13.3)	12.8 (11.0–14.36)	0.499
D-dimer (ug/mL)	2587 (950–10,000)	6139 (1644–10,000)	1264 (788–5535)	< 0.001
Fibrinogen (g/L)	7.0 (6.0–10.0)	6.9 (6.0–9.6)	7.4 (6.0–10.0)	0.441
**Thromboelastography parameters (*n*** **= 20) (normal ranges)**	*n* = 20	*n* = 12	*n* = 8	
R time (mins) (4.6–9.1)	7.37 (2.45)	7.70 (1.87)	6.86 (3.22)	0.094
CK alpha (angle) (63–78)	75.7 (3.4)	75.5 (3.5)	76.1 (3.3)	0.156
MA (mm) (52–69)	69.3 (2.26)	69.3 (1.70)	69.4 (3.06)	0.169
Platelet contribution to clot strength (%)	64 (61–73)	63 (53–69)	68 (64–77)	0.552
Fibrin contribution to clot strength (%)	36 (27–39)	37 (31–47)	32 (23–36)	–
Thrombodynamic ratio	17.1 (11.5–24.6)	14.1 (11.5–18.0)	24.5 (13.9–24.8)	0.201
LY30 (0.0–2.6)	0.00 (0.00–0.05)	0.00 (0.00–0.00)	0.00 (0.00–0.48)	0.240
**Outcome as of 15 May 2020**, *n* (%)				
Died in ICU	59 (31.6)	32 (39.5)	27 (25.5)	0.059
Still alive in ICU	33 (17.6)	17 (21.0)	16 (15.1)	–
Discharged alive from ICU	95 (50.8)	32 (39.5)	63 (59.4)	–
**ICU length of stay (days)**, median (IQR)	15 (7–21)	17 (11–27)	12 (7–13)	0.003

*APACHE* Acute Physiology and Chronic Health Evaluation, *DIC* disseminated Intravascular coagulation, *ICU* intensive care unit, *LWMH* low molecular weight heparin

## Discussion

### Key results

Our multicentre study supports previous reports of a high incidence of thromboembolic complications in ICU patients with COVID-19, despite the initiation of thromboprophylaxis [7–10]. We observed a higher incidence of arterial ischaemic and bleeding complications when compared with previous COVID-19 and non-COVID ICU data [7, 8, 21]. Laboratory parameters linked to thromboinflammation, such as D-dimer, white cell count, troponin and ferritin were higher in patients who developed thrombotic complications. Thromboelastography, however, lacked discriminatory value.

### Interpretation

The high burden of thrombotic complications in COVID-19, combined with data from other viral respiratory illnesses [22], has led to international clinical guidelines now recommending higher doses (intermediate dose) of thromboprophylaxis [13]. However, a consensus statement written on behalf of the American College of Cardiology recommends against the use of the intermediate dose LWMH in patients with moderate-severe COVID-19 [11].

Observational data, with limited control for confounders, have provided conflicting results on the mortality benefit associated with anticoagulation in COVID-19. Tang et al. [23] in a single-centre study demonstrated a reduction in 28-day mortality with heparin treatment. However, it was conducted in a setting where routine thromboprophylaxis is not standard practice. Paranjpe et al. [24], in a database study involving 2773 hospitalised COVID-19 patients, showed improved survival with systemic anticoagulation. However, there was no survival difference in mechanically ventilated patients and details on the pharmacological agent used and dose were not provided.

Given the strong procoagulant component that accompanies the pathophysiology of COVID-19, one hypothesis that has generated interest is whether being on anticoagulation for unrelated conditions prior to COVID-19 is protective for COVID-19 related outcomes. A recent propensity score-matched study compared 913 patients receiving anticoagulation (antiplatelet or anticoagulation therapy) with 2859 patients receiving neither at the time of COVID-19 diagnosis and found no statistically significant difference in survival, mechanical ventilation and need for hospital admission between both groups [25]. The uncertainty highlighted in observational studies and disagreement between guidelines re-iterates the importance of ongoing randomised trials such as COVID-HEP (NCT04345848) and REMAP-CAP [26].

Rather concerningly, we report a higher incidence of arterial ischaemic complications when compared with previous studies. Helms et al. only reported four (2.6%) cases in a cohort study of 150 COVID-19 ICU patients [7]. Arterial complications may result in major morbidity as highlighted in recent case reports [27, 28]. It is unclear whether the same mechanisms are involved in venous as in arterial clots. Arterial clots in COVID-19 patients removed at the time of surgery [28] have been consistent with being platelet-rich which suggests that anti-platelet agents may have a role to play in preventing arterial complications.

Increased doses of thromboprophylaxis must be carefully weighed against the risks of bleeding. These risks are not insubstantial in critically ill patients. Rates of gastrointestinal haemorrhage among general ICU patients have been described as approximately 3.5% [21], with increasing bleeding risk in sicker patients and those with predisposing illness, such as liver or haematological disease. In an international multicentre study enrolling 3746 critically ill patients receiving heparin thromboprophylaxis, bleeding occurred in 5.6% of patients [29]. Gastrointestinal bleeding was the commonest site occurring in 51.9% of patients (2.9% of the overall cohort). Factors associated with bleeding included prolonged activated partial thromboplastin time, thrombocytopenia, therapeutic heparin anticoagulation, antiplatelet therapy, renal replacement therapy and recent surgery. The type of agent used for thromboprophylaxis was not associated with bleeding.

Previously reported rates of bleeding in COVID-19 range from 0 to 7.5% [7, 8, 23] and are poorly described with regard to clinical characteristics, timing and anatomical site. A recent study of 400 COVID-19 patients, Al-Samkari et al. reported an overall bleeding rate of 7.6% in critically ill patients, with a major bleeding rate of 5.6% [30]. These data, combined with our findings, suggest that empiric increases in thromboprophylaxis doses beyond standard care should be pursued with caution. Patients who experienced bleeding complications in our cohort were predominantly male, with higher SOFA scores and ICU resource requirements. We observed that bleeding was not associated with deranged laboratory evidence of coagulopathy, but it is recognised that markers such as prothrombin time are poor predictors of bleeding [31]. The overall rate of clinically important gastrointestinal bleeding in our cohort was 3.2% which is in keeping with non-COVID ICU patients [21, 29]. Our data suggest that even in the absence of laboratory evidence of coagulopathy in COVID-19, clinicians should maintain a high index of suspicion of bleeding. The variation in bleeding rates reported in COVID-19 also highlights the need to standardise assessment of bleeding in order to allow for better comparison between studies.

The predominant site of bleeding linked to COVID-19 is intrapulmonary microhaemorrhage, and it may be that pulmonary intravascular coagulopathy eventually progresses to systemic coagulopathy [32]. Laboratory evidence of coagulopathy was also rare during the SARS-CoV-1 outbreak in 2002 [33]. In contrast, haemorrhage has been frequently reported with other viral infections such as Ebola [34], where organ damage is predominantly in the liver and peripheral vascular beds, which suggests the dominance of either bleeding or thrombosis depends on the causal virus. Therefore, whether our findings represent a true increase in bleeding risk directly due to immune mechanisms related to COVID-19, anticoagulation or are as a result of illness severity remains unclear. Gastrointestinal and intracranial bleeding are also recognised complications when anticoagulation is necessary to facilitate rescue treatments such as extracorporeal membrane oxygenation [35].

Elevated concentrations of D-dimer, ferritin, troponin and white cell count at ICU admission may reflect undiagnosed clot burden prior to admission and be used to identify patients for CTPA. Various D-Dimer thresholds have been proposed but these have poor sensitivity ranging from 67 to 70% [36, 37]. It is unclear whether these high values are as a direct consequence of SARS-CoV-2 itself or if this is secondary to systemic inflammation secondary to critical illness. Thromboelastography may be a useful tool for guiding therapy in major haemorrhage [38]. Our data demonstrated a hypercoagulable profile with little or no fibrinolysis in keeping with previously published data [39, 40], although disappointingly it did not discriminate between those with and without thrombotic complications. However, data were only available for twenty patients in our study. Prospective studies investigating its use to assess bleeding and thrombotic risk and guide anticoagulation in COVID-19 are needed [1]. At present, we do not advocate using thromboelastography results to guide clinical management outside of prospective clinical research.

### Strengths and limitations

Strengths of our work include the largest reported multicentre cohort evaluating thrombotic complications in critically ill patients, completeness of the data, and for the first time to our knowledge, a detailed description of bleeding complications. Our study cohort is also representative of our national population. Limitations of our study include underreporting, and therefore, the rates of complications could be higher than reported here. We lacked a comparator non-COVID ICU patient group, but these data have been reported previously [7] and are unlikely to alter the conclusions of our findings.

## Conclusion

Critically ill patients with COVID-19 experience high rates of thrombotic complications. Our data suggest that clinicians should consider that bleeding and arterial ischaemic complications may also be more frequent in these patients. Our work re-iterates the need for randomised trials to better understand the risk-benefit ratio of different anticoagulation strategies.

## Supplementary information

**Additional file 1.** STROBE checklist of items that should be reported in observational studies.

**Additional file 2.** Thromboprophylaxis protocols for each participating site.

**Additional file 3.** Thrombotic and haemorrhagic complications by individual participating centre.

**Additional file 4.** Sensitivity analysis of study cohort with laboratory confirmed SARS-CoV-2 and cohort with confirmed and suspected SARS-CoV-2 infection.

## Data Availability

The dataset used and analysed for this study are available from the corresponding author on reasonable request.

## Abbreviations

COVID-19: Coronavirus disease 2019
CTPA: Computerised tomography pulmonary angiogram
DIC: Disseminated intravascular coagulation
ICU: Intensive care unit
IQR: Interquartile range
LMWH: Low molecular weight heparin
RT-PCR: Reverse-transcriptase polymerase chain reaction
SARS-CoV-1: Severe acute respiratory coronavirus 1
SARS-CoV-2: Severe acute respiratory coronavirus 2
SOFA: Sequential Organ Failure Assessment
STROBE: Strengthening the Reporting of Observational Studies in Epidemiology

## References

[1] Joly BS, Siguret V, Veyradier A. Understanding pathophysiology of hemostasis disorders in critically ill patients with COVID-19. Intensive Care Med. 2020. 10.1007/s00134-020-06088-1. 10.1007/s00134-020-06088-1 PMC7225398 32415314

[2] Mojoli F, Mongodi S, Orlando A, Arisi E, Pozzi M, Civardi L (2020). Our recommendations for acute management of COVID-19. Crit Care.

[3] Murthy S, Gomersall CD, Fowler RA (2020). Care for critically ill patients with COVID-19. JAMA..

[4] Ackermann M, Verleden SE, Kuehnel M, Haverich A, Welte T, Laenger F, et al. Pulmonary vascular endothelialitis, thrombosis, and angiogenesis in Covid-19. N Engl J Med. 2020. 10.1056/NEJMoa2015432. 10.1056/NEJMoa2015432 PMC7412750 32437596

[5] Wang Y, Lu X, Chen H, Chen T, Su N, Huang F (2020). Clinical course and outcomes of 344 intensive care patients with COVID-19. Am J Respir Crit Care Med.

[6] Wang D, Yin Y, Hu C, Liu X, Zhang X, Zhou S (2020). Clinical course and outcome of 107 patients infected with the novel coronavirus, SARS-CoV-2, discharged from two hospitals in Wuhan. China Crit Care.

[7] Helms J, Tacquard C, Severac F, Leonard-Lorant I, Ohana M, Delabranche X, et al. High risk of thrombosis in patients with severe SARS-CoV-2 infection: a multicenter prospective cohort study. Intensive Care Med. 2020. 10.1007/s00134-020-06062-x. 10.1007/s00134-020-06062-x PMC7197634 32367170

[8] Klok FA, Kruip M, van der Meer NJM, Arbous MS, Gommers D, Kant KM (2020). Incidence of thrombotic complications in critically ill ICU patients with COVID-19. Thromb Res.

[9] Di Micco P, Russo V, Carannante N, Imparato M, Rodolfi S, Cardillo G (2020). Clotting factors in COVID-19: epidemiological association and prognostic values in different clinical presentations in an Italian cohort. J Clin Med.

[10] Llitjos JF, Leclerc M, Chochois C, Monsallier JM, Ramakers M, Auvray M, et al. High incidence of venous thromboembolic events in anticoagulated severe COVID-19 patients. J Thromb Haemost. 2020. 10.1111/jth.14869. 10.1111/jth.14869 PMC7264774 32320517

[11] Fraissé M, Logre E, Pajot O, Mentec H, Plantefeve G, Contou D (2020). Thrombotic and hemorrhagic events in critically ill COVID-19 patients: a French monocenter retrospective study. Crit Care.

[12] Bikdeli B, Madhavan MV, Jimenez D, Chuich T, Dreyfus I, Driggin E, et al. COVID-19 and thrombotic or thromboembolic disease: implications for prevention, antithrombotic therapy, and follow-up. J Am Coll Cardiol. 2020. 10.1016/j.jacc.2020.04.031. 10.1016/j.jacc.2020.04.031 PMC7164881 32311448

[13] Spyropoulos AC, Levy JH, Ageno W, Connors JM, Hunt BJ, Iba T, et al. Scientific and standardization committee communication: clinical guidance on the diagnosis, prevention and treatment of venous thromboembolism in hospitalized patients with COVID-19. J Thromb Haemost. 2020. 10.1111/jth.14929. 10.1111/jth.14929 PMC7283841 32459046

[14] Susen S, Tacquard CA, Godon A, Mansour A, Garrigue D (2020). Prevention of thrombotic risk in hospitalized patients with COVID-19 and hemostasis monitoring. Crit Care.

[15] von Elm E, Altman DG, Egger M, Pocock SJ, Gotzsche PC, Vandenbroucke JP (2007). The Strengthening the Reporting of Observational Studies in Epidemiology (STROBE) statement: guidelines for reporting observational studies. Lancet..

[16] Witt DM, Nieuwlaat R, Clark NP, Ansell J, Holbrook A, Skov J (2018). American Society of Hematology 2018 guidelines for management of venous thromboembolism: optimal management of anticoagulation therapy. Blood Adv.

[17] Intensive Care National Audit and Research Centre. COVID-19 report. 2020. https://www.icnarc.org/Our-Audit/Audits/Cmp/Reports. Accessed 24 May 2020.

[18] Schulman S, Kearon C, Subcommittee on Control of Anticoagulation of the S, Standardization Committee of the International Society on T, Haemostasis Definition of major bleeding in clinical investigations of antihemostatic medicinal products in non-surgical patients J Thromb Haemost 2005;3:692–694. 10.1111/j.1538-7836.2005.01204.x 15842354

[19] Taylor FB, Jr., Toh CH, Hoots WK, Wada H, Levi M, Scientific Subcommittee on Disseminated Intravascular Coagulation of the International Society on T, et al. Towards definition, clinical and laboratory criteria, and a scoring system for disseminated intravascular coagulation. Thromb Haemost. 2001;86:1327–1330. 11816725

[20] Rockwood K, Song X, MacKnight C, Bergman H, Hogan DB, McDowell I (2005). A global clinical measure of fitness and frailty in elderly people. CMAJ..

[21] Cook DJ, Griffith LE, Walter SD, Guyatt GH, Meade MO, Heyland DK (2001). The attributable mortality and length of intensive care unit stay of clinically important gastrointestinal bleeding in critically ill patients. Crit Care.

[22] Obi AT, Tignanelli CJ, Jacobs BN, Arya S, Park PK, Wakefield TW (2019). Empirical systemic anticoagulation is associated with decreased venous thromboembolism in critically ill influenza A H1N1 acute respiratory distress syndrome patients. J Vasc Surg Venous Lymphat Disord.

[23] Tang N, Bai H, Chen X, Gong J, Li D, Sun Z (2020). Anticoagulant treatment is associated with decreased mortality in severe coronavirus disease 2019 patients with coagulopathy. J Thromb Haemost.

[24] Paranjpe I, Fuster V, Lala A, Russak A, Glicksberg BS, Levin MA, et al. Association of treatment dose anticoagulation with in-hospital survival among hospitalized patients with COVID-19. J Am Coll Cardiol. 2020. 10.1016/j.jacc.2020.05.001. 10.1016/j.jacc.2020.05.001 PMC7202841 32387623

[25] Tremblay D, van Gerwen M, Alsen M, Thibaud S, Kessler AJ, Venugopal S, et al. Impact of anticoagulation prior to COVID-19 infection: a propensity score-matched cohort study. Blood. 2020. 10.1182/blood.2020006941. 10.1182/blood.2020006941 PMC7332896 32462179

[26] Angus DC, Berry S, Lewis RJ, Al-Beidh F, Arabi Y, van Bentum-Puijk W, et al. The Randomized Embedded Multifactorial Adaptive Platform for Community-acquired Pneumonia (REMAP-CAP) study: rationale and design. Ann Am Thorac Soc. 2020. 10.1513/AnnalsATS.202003-192SD. 10.1513/AnnalsATS.202003-192SD PMC7328186 32267771

[27] Azouz E, Yang S, Monnier-Cholley L, Arrive L. Systemic arterial thrombosis and acute mesenteric ischemia in a patient with COVID-19. Intensive Care Med. 2020; 10.1007/s00134-020-06079-2. 10.1007/s00134-020-06079-2 PMC7232609 32424482

[28] Griffin DO, Jensen A, Khan M, Chin J, Chin K, Parnell R, et al. Arterial thromboembolic complications in COVID-19 in low-risk patients despite prophylaxis. Br J Haematol. 2020. 10.1111/bjh.16792.. 10.1111/bjh.16792 PMC7267572 32374029

[29] Al-Samkari H, Leaf RK, Dzik WH, Carlson JCT, Fogerty AE, Waheed A, et al. COVID and coagulation: bleeding and thrombotic manifestations of SARS-CoV2 infection. Blood. 2020. 10.1182/blood.2020006520. 10.1182/blood.2020006520 PMC7378457 32492712

[30] Shah A, Stanworth SJ, McKechnie S (2015). Evidence and triggers for the transfusion of blood and blood products. Anaesthesia.

[31] McGonagle D, O’Donnell JS, Sharif K, Emery P, Bridgewood C (2020). Immune mechanisms of pulmonary intravascular coagulopathy in COVID-19 pneumonia. Lancet Rheum.

[32] Wong RSM, Wu A, Lee N, Lam CWK, Wong CK, To KF (2003). Haematological manifestations in patients with severe acute respiratory syndrome: retrospective analysis. BMJ.

[33] Feldmann H, Geisbert TW (2011). Ebola haemorrhagic fever. Lancet.

[34] Kowalewski M, Fina D, Slomka A, Raffa GM, Martucci G, Lo Coco V (2020). COVID-19 and ECMO: the interplay between coagulation and inflammation-a narrative review. Crit Care.

[35] Leonard-Lorant I, Delabranche X, Severac F, Helms J, Pauzet C, Collange O, et al. Acute pulmonary embolism in COVID-19 patients on CT angiography and relationship to D-dimer levels. Radiology. 2020;201561. 10.1148/radiol.2020201561 PMC7233397 32324102

[36] Cui S, Chen S, Li X, Liu S, Wang F. Prevalence of venous thromboembolism in patients with severe novel coronavirus pneumonia. J Thromb Haemost. 2020. 10.1111/jth.14830. 10.1111/jth.14830 PMC7262324 32271988

[37] Spahn DR, Bouillon B, Cerny V, Duranteau J, Filipescu D, Hunt BJ (2019). The European guideline on management of major bleeding and coagulopathy following trauma: fifth edition. Crit Care.

[38] Panigada M, Bottino N, Tagliabue P, Grasselli G, Novembrino C, Chantarangkul V, et al. Hypercoagulability of COVID-19 patients in intensive care unit. A report of thromboelastography findings and other parameters of hemostasis. J Thromb Haemost. 2020; 10.1111/jth.14850. 10.1111/jth.14850 PMC9906150 32302438

[39] Ranucci M, Ballotta A, Di Dedda U, Bayshnikova E, Dei Poli M, Resta M, et al. The procoagulant pattern of patients with COVID-19 acute respiratory distress syndrome. J Thromb Haemost. 2020. 10.1111/jth.14854. 10.1111/jth.14854 PMC9906332 32302448

[40] Lauzier F, Arnold DM, Rabbat C, Heels-Ansdell D, Zarychanski R, Dodek P (2013). Risk factors and impact of major bleeding in critically ill patients receiving heparin thromboprophylaxis. Intensive Care Med.

