# Supplementary material for: Thrombotic and haemorrhagic complications in critically ill patients with COVID-19: a multicentre observational study
Source: Crit Care. 2020 Sep 18;24:561. doi: 10.1186/s13054-020-03260-3 (PMC7499016; doi:10.1186/s13054-020-03260-3)
Supplement: Supplementary file 2 — Additional file 2. Thromboprophylaxis protocols for each participating site. [file 13054_2020_3260_MOESM2_ESM.docx]

**Additional File 2: Thromboprophylaxis (subcutaneous) protocols at each participating site**

**John Radcliffe Hospital, Oxford (Site 1)**

*Pre-COVID/early COVID*

| **Weight (kg)** | **Dalteparin dose (units)** |
| --- | --- |
| Less than 46 | 2500 once daily |
| 46-120 | 5000 once daily |
| 121-150 | 7500 once daily |
| More than 150 | 5000 twice daily |

*During COVID – changed to on 24^th^ April 2020*

| **Weight (kg)** | **Dalteparin dose (units)** |
| --- | --- |
| Less than 46 | 2500 twice daily |
| 46-120 | 5000 twice daily |
| 121-150 | 7500 twice daily |
| More than 150 | 10,000 twice daily |

For patients with severe renal impairment (eGFR <20 ml/min/1.73 m^2^), anti-Xa levels were checked after 3-4 days targeting a peak level in the region of 0.3 to 0.4 heparin anti-Xa units/mL with trough levels between 0.0 to 0.2 anti Xa units/mL.

**Bristol Royal Infirmary (Site 2) and Southmead Hospital (Site 3), Bristol**

*Pre-COVID/early COVID* (if eGFR >30 ml/min/1.73 m^2^)

| **Weight (kg)** | **Enoxaparin** |
| --- | --- |
| Less than 50 | 20mg once daily |
| 50 - 100 | 40mg once daily |
| More than 100 | 40mg twice daily |

*During COVID –switched on 28^th^ March 2020 (Southmead Hospital) and 7^th^ April 2020 (Bristol Royal Infirmary)*

Normal renal function (if eGFR >30 ml/min/1.73 m^2^)

| **Weight (kg)** | **Enoxaparin** |
| --- | --- |
| Less than 50 | 20mg twice daily |
| 50 - 100 | 40mg twice daily |
| More than 100 | 60mg twice daily |

Impaired renal function (if eGFR <30 ml/min/1.73 m^2^)

| **Weight (kg)** | **Unfractionated heparin (units)** |
| --- | --- |
| Less than 50 | 2500 IU twice daily |
| 50 - 100 | 5000 IU twice daily |
| More than 100 | 5000 IU thrice daily |

**Cardiff and Vale University Health Board (Site 4), Wales**

*Pre-COVID/early COVID*

| **Creatine clearance** | **≥ 30 ml/min** | **15-29 ml/min** |
| --- | --- | --- |
| **Weight (kg)** | **Enoxaparin** | **Unfractionated heparin (units)** |
| Less than 50 | 20mg once daily | 5000 IU twice daily |
|  |  | **Enoxaparin** |
| 50-99 | 40mg once daily | 20mg once daily |
| 100-149 | 40mg twice daily | 40mg once daily |
| ≥ 150 | 60mg twice daily | 40mg once daily |

*During COVID – changed to on 22^th^ April 2020*

| **Creatine clearance** | **≥ 30 ml/min** | **15-29 ml/min** |
| --- | --- | --- |
| **Weight (kg)** | **Enoxaparin** | **Enoxaparin** |
| Less than 50 | 20mg twice daily | 20mg once daily |
| 51-75 | 40mg twice daily | 20mg twice daily |
| 76-100 | 60mg twice daily | 20mg twice daily |
| 101-125 | 80mg twice daily | 40mg twice daily |
| 125-150 | 100mg twice daily | 40mg twice daily |
| >151 | 120mg twice daily | 40mg twice daily |
