# Supplementary material for: Thrombotic and haemorrhagic complications in critically ill patients with COVID-19: a multicentre observational study
Source: Crit Care. 2020 Sep 18;24:561. doi: 10.1186/s13054-020-03260-3 (PMC7499016; doi:10.1186/s13054-020-03260-3)
Supplement: Supplementary file 3 — Additional file 3. Thrombotic and haemorrhagic complications by individual participating centre. [file 13054_2020_3260_MOESM3_ESM.docx]

**Additional File 3.** Thrombotic and bleeding complications by study site

|  | **Total (n=187)** | **Oxford (n = 51)** | **Bristol Royal Infirmary (n = 25)** | **North Bristol NHS Trust (n = 40)** | **Cardiff (n = 71)** |
| --- | --- | --- | --- | --- | --- |
| **Thrombotic complications,** n (%)  Pulmonary embolism  Deep vein thrombosis  Arterial complications:   - Arterial ischaemia (peripheral or intestinal) - Cerebrovascular accident - Myocardial infarction   Extracorporeal circuit disruption | **81 (43.3)** | **25 (49.0)** | **17 (68.0)** | **10 (25.0)** | **29 (40.8)** |
|  | 42 (22.5) | 14 (27.5) | 10 (40.0) | 6 (15.0) | 12 (16.9) |
|  | 22 (11.8) | 12 (23.5) | 6 (24.0) | 1 (2.5) | 3 (4.2) |
|  | 12 (6.4) | 0 (0.0) | 2 (8.0) | 2 (5.0) | 8 (11.3) |
|  | 8 (4.3) | 2 (3.9) | 1 (4.0) | 1 (2.5) | 4 (5.6) |
|  | 5 (2.7) | 0 (0.0) | 2 (8.0) | 0 (0.0) | 3 (4.2) |
|  | 23 (12.3) | 8 (15.7) | 8 (16.0) | 1 (2.5) | 6 (8.5) |
| **Bleeding complications,** n (%)  Intracranial  Gastrointestinal  Other (Epistaxis (n=1), Tracheostomy (n=1),   Genito-Urinary (n=2)) | **15 (8.0)** | **8 (15.7)** | **2 (8.0)** | **4 (10.0)** | **1 (1.4)** |
|  | 5 (2.7) | 1 (2.0) | 1 (4.0) | 2 (5.0) | 1 (1.4) |
|  | 6 (3.2) | 5 (9.8) | 1 (4.0) | 0 (0.0) | 0 |
|  | 4 (2.1) | 2 (3.9) | 0 (0.0) | 2 (5.0) | 0 |
