# Supplementary material for: Thrombotic and haemorrhagic complications in critically ill patients with COVID-19: a multicentre observational study
Source: Crit Care. 2020 Sep 18;24:561. doi: 10.1186/s13054-020-03260-3 (PMC7499016; doi:10.1186/s13054-020-03260-3)
Supplement: Supplementary file 4 — Additional file 4. Sensitivity analysis of study cohort with laboratory confirmed SARS-CoV-2 and cohort with confirmed and suspected SARS-CoV-2 infection. [file 13054_2020_3260_MOESM4_ESM.docx]

**Additional File 4.** Sensitivity analysis of study cohort with laboratory confirmed SARS-CoV-2 and cohort with confirmed and suspected SARS-CoV-2 infection

|  | Laboratory confirmed only (*n* = 170) | Total cohort (Laboratory confirmed + suspected COVID-19) (*n* = 187) |
| --- | --- | --- |
| **No. of patients with thrombotic complications**, n (%)  Pulmonary embolism  Deep vein thrombosis  Arterial complications   - Arterial ischaemia (peripheral or intestinal) - Cerebrovascular accident - Myocardial infarction   Extracorporeal circuit disruption | 71 (41.7)  32 (18.8)  20 (11.7)  12 (7.0)  8 (4.7)  5 (2.9)  22 (12.9) | 81 (43.3)  44 (22.5)  22 (11.8)  12 (6.4)  8 (4.3)  5 (2.7)  23 (12.3) |
| **Bleeding**, n (%) | 12 (7.0%) | 15 (8.0%) |
